# Recrystallization Mediates the Gelation of Amorphous Drugs: The Case of Acemetacin

**DOI:** 10.3390/pharmaceutics15010219

**Published:** 2023-01-08

**Authors:** Manlin Teng, Jianfeng Li, Zhaohua Li, Guangshuai Zhang, Peixu Zhao, Qiang Fu

**Affiliations:** Wuya College of Innovation, Shenyang Pharmaceutical University, No. 103, Wenhua Road, Shenyang 110016, China

**Keywords:** acemetacin, amorphous, gelation, recrystallization

## Abstract

Amorphization is widely used as an effective method of increasing the solubility of insoluble drugs. However, some amorphous drugs exhibit a much lower dissolution rate than their corresponding crystalline form due to their gelation. In this study, we reported the gels formed from amorphous acemetacin (ACM) for the first time. Gelation was promoted at conditions of lower pH, higher temperature and lower ionic strength. Solid-state characterizations suggested that ACM gels may be formed by recrystallization. This mechanism provides a new direction in facilitating the elimination of gelation for amorphous drugs. Moreover, it also provides the basis for the development of sustained-release formulations using the gelation properties.

## 1. Introduction

At present, more than 70% of the drugs in the development pipeline have poor solubility [1] which hinders their oral bioavailability [2,3]. These drugs typically have an ordered structure with a high lattice energy that is difficult to disrupt [4]. Amorphization can improve the dissolution efficiently, because high surface free energy can be obtained by changing the original long-range ordered structure in the crystalline structure to a disordered one [5]. To date, many amorphous formulations have been developed for the improvement of oral bioavailability for poorly water-soluble drugs [6,7].

Neat amorphous drugs are an attractive method for achieving 100% drug loading without the addition of any excipients [8]. However, some neat amorphous drugs, such as lurasidone [9] and curcumin [10], show much lower dissolution rates than the corresponding crystalline form due to gelation. At present, the main explanation for crystalline gels is the formation of a supercooled state due to low glass transition temperature (Tg) promoting drug recrystallization and gelation [10,11]. However, some studies have found that reduced solubility can also lead to gelation of drugs [12]. Although recrystallization-mediated gel formation has been reported for amorphous drugs [9,10], this mechanism still faces challenges. Thus, it is necessary to further study the formation mechanism of amorphous drug gels.

Acemetacin (ACM), a non-steroidal anti-inflammatory drug, belongs to biopharmaceutics classification system class VI and is virtually insoluble in water with a solubility of 2.29 µg/mL. It has a melting point of 151.5 °C and a decomposition temperature of 271 °C [13]. In this study, we report the gels formed from amorphous ACM for the first time. The amorphous ACM prepared by the melt quenching method showed a much slower dissolution rate than the crystalline counterpart because the amorphous ACM powders rapidly gelled upon hydration. To explore the gelation mechanism, the formed gel was characterized by polarizing light microscopy, differential scanning calorimetry, X-ray powder diffractometry, Fourier transform infrared spectroscopy and a texture analyzer. Then, we attempted to probe the internal relationship between gelation and hydration. The exploration of gelation mechanisms of amorphous drugs would provide insights for inhibition of the amorphous drug gelation and provide a basis for the development of poorly water-soluble drugs.

## 2. Materials and Methods

### 2.1. Materials

ACM was provided by Hubei Huizepu Medical Technology Co., Ltd. (Wuhan, China). Sodium chloride (NaCl) was provided by Tianjin Hengxing Chemical Reagent Co., Ltd. (Tianjin, China). Potassium bromide (KBr) was purchased from Sinopharm Chemical Reagent Co., Ltd. (Shanghai, China). All other reagents and chemicals were of analytical grade.

### 2.2. Preparation of Amorphous ACM

Amorphous ACM was prepared using the melt quenching method. Typically, ACM (500 mg) was heated at 160 °C in an aluminum dish until it was completely melted, and then quickly subjected to quench cooling in liquid nitrogen. The resultant solid was gently grounded with a mortar and sieved through an 80-meshed sieve, and then it was stored in a vacuum desiccator overnight for further studies.

### 2.3. Dissolution Tests

#### 2.3.1. Dissolution under Non-Sink Conditions

To explore the dissolution behavior of ACM, dissolution tests were conducted using a ZRS-8G dissolution apparatus (Tianda Tianfa Technology Co., Ltd., Tianjin, China) according to Chinese Pharmacopeia Apparatus II method (paddle method). The studies were performed in 900 mL of deionized water at 37 °C. About 30 mg of ACM (crystalline or amorphous) was added in the dissolution media, and the rotating speed was set at 100 rpm. At pre-set time points (5, 10, 20, 30 and 60 min), 10 mL of the medium was withdrawn and replaced with an equal volume of fresh water. After filtered through a 0.45 µm filter membrane, the subsequent filtrate was quantified at a wavelength of 319 nm using a UV-1102II ultraviolet spectrophotometer (Techcomp (China) Ltd., Shanghai China). After dissolution, the formed gels from amorphous ACM were collected and dried for solid state characterization as described in Section 2.4.

#### 2.3.2. Intrinsic Dissolution Rate

The intrinsic dissolution rate (IDR) of the crystalline and amorphous ACM were tested using a RC301 dissolution apparatus (Tianfa Analysis Instrument Co., Ltd., China). Disks were obtained by pressing 100 mg of each sample in radius 4 mm dies at 50 bar for 30 s. The disks were held in 500 mL of deionized water and the rotation speed of the disk was 100 rpm. The temperature of the dissolution instrument was kept at 37 °C. Five milliliters of samples were removed at predetermined time points (5, 10, 20, 30 and 60 min) and refreshed with an equal volume of fresh water. Then, the samples were filtered using 0.45 µm filter membranes. ACM concentrations were determined using the UV method as described in Section 2.3.1.

### 2.4. Solid-State Characterization

#### 2.4.1. Polarizing Light Microscopy

Polarizing light microscopy (PLM) images were obtained using a DM2700P polarizing light microscope (Leica Microsystems Ltd., Weztlar, Germany). About 10 mg of amorphous ACM was placed on a slide and dispersed evenly. Then, 50 µL of water was added to the powder and the recrystallization was observed under PLM at different time points (0, 15, 30, 45, 60 and 75 s). The PLM images were captured at 10X magnification under a dark field.

#### 2.4.2. Differential Scanning Calorimetry

The thermal characteristics of samples were collected using a DSC1 apparatus (Mettler-Toledo International Inc., Schwerzenbach, Switzerland). The aluminum oxide crucible was selected to calibrate the temperature and cell constants. About 6 mg of each sample was sealed in a standard aluminum crucible and heated to 180 °C at a rate of 10 °C/min. After that, the samples were cooled to 0 °C at 30 °C/min and balanced for 1 min, then they were reheated to 180 °C at a rate of 10 °C/min to measure the Tg. All procedures were carried out under nitrogen protection.

#### 2.4.3. X-Ray Powder Diffractometry

X-ray powder diffractometry (XRPD) analyses were performed using a Dmax-2500 Powder X-ray diffractometer (Rigaku Corporation, Tokyo, Japan). The instrument was operated at 40 kV and 40 mA. The samples were scanned from 5−40° at a scanning speed of 5°/min with a step size of 0.01°.

#### 2.4.4. Fourier-Transform Infrared Spectroscopy

Fourier-transform infrared spectroscopy (FTIR) spectra were obtained by the potassium bromide disc technique using an IFS 55 FT-IR spectrometer (Bruker Ltd., MA, Switzerland). Each sample was scanned from 4000 to 400 cm^−1^ and resolution was taken as 4 cm^−1^.

### 2.5. Influencing Factors of Amorphous ACM Gelation

Gel formation experiments were performed in different media, with the purpose to investigate the effects of temperature, pH and ionic strength on the gelation properties of amorphous ACM. Firstly, tests were carried out in water at different temperatures (20, 30, 37 and 45 °C) to evaluate the relationship between the temperature and gelation. Additional tests were conducted in different buffers (pH 1.2, 4.5, 6.8 and 7.4) at room temperature. Tests to examine the effect of ionic strength were conducted in NaCl solutions (0.25, 0.5, 0.75 and 1 M) at 37 °C. Amorphous ACM (about 500 mg) was added to 10 mL of media and stirred uniformly with a glass rod until the gel was formed. The gelation time was recorded. Then, the formed gel was taken out for texture profile analysis below.

### 2.6. Texture Profile Analysis

After dissolution, the ACM gels were taken out and water on the surface was removed immediately. Then, the gelling characteristics (adhesiveness, cohesiveness, and springiness) were evaluated using a CT3 texture analyzer (Brookfield, Middleboro, MA, USA). The samples were placed carefully on a fixed base (TA-BT-KIT). Then, they were compressed with a trigger force of 5.0 g under a cylindrical probe (P/0.5R). The pre-test speed, test speed and post-test speed were set to 1.0, 5.0 and 5.0 mm/s, respectively. All measurements were conducted in triplicate.

## 3. Results and Discussion

### 3.1. Dissolution Behaviors of Crystalline and Amorphous ACM

**Figure 1:**
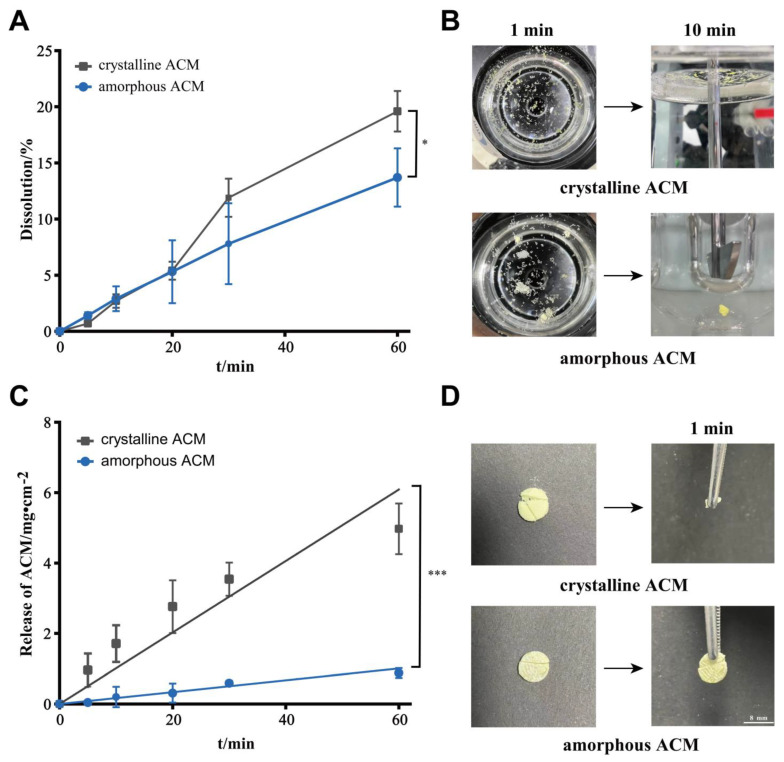
(**A**) Dissolution profiles and (**C**) IDR curves of crystalline ACM and amorphous ACM in water at 37 °C. Results are expressed as the mean ± SD (*n* = 3, * *p* < 0.05 and *** *p* < 0.001 vs. crystalline ACM). (**B**) Photographs of crystalline ACM and amorphous ACM in a glass vessel during dissolution in water at 1 min (vertical viewing) and 10 min (horizontal viewing). (**D**) Photographs of crystalline ACM and amorphous ACM after intrinsic dissolution in 2 min.

The dissolution profiles of crystalline and amorphous ACM in water at 37 °C are shown in Figure 1A. At the beginning of dissolution, the amorphous ACM showed higher concentrations than the crystalline counterpart, because amorphous phases lack the long-range order and periodicity of crystalline states and have high free energy and low density [14]. However, it was noticed that the dissolution rate of amorphous ACM slowed down after 10 min and was lower than that of crystalline ACM due to its gelation [11,15]. As shown in Figure 1B, crystalline ACM floated on the surface of the medium. Nevertheless, the dispersed amorphous ACM powder spontaneously aggregated in 1 min and floated on the liquid surface to form a layer of “film”, and it further agglomerated and formed a thick and sticky gel in 10 min.

Intrinsic dissolution can clearly reflect the dissolution characteristics of a drug under a constant surface area, and it is an effective method for the evaluation of aqueous solubility for polymorphic drugs [16,17]. As shown in Figure 1D, the ACM disk was first cut into halves, and then the two halves were placed in contact. The results show that the amorphous disk was gelled in 1 min and adhered together, whereas the crystalline ACM showed no gelation and still separated. The IDR curve showed that the gelation inhibited the dissolution of amorphous ACM (Figure 1C). The IDR of amorphous ACM (17.86 ± 2.19 µg⋅cm^−2^⋅min^−1^) was significantly lower (*p* < 0.001) than that of crystalline ACM (75.66 ± 10.83 µg⋅cm^−2^⋅min^−1^). In summary, amorphous ACM lost its dissolution advantage after gelation.

### 3.2. PLM

**Figure 2:**
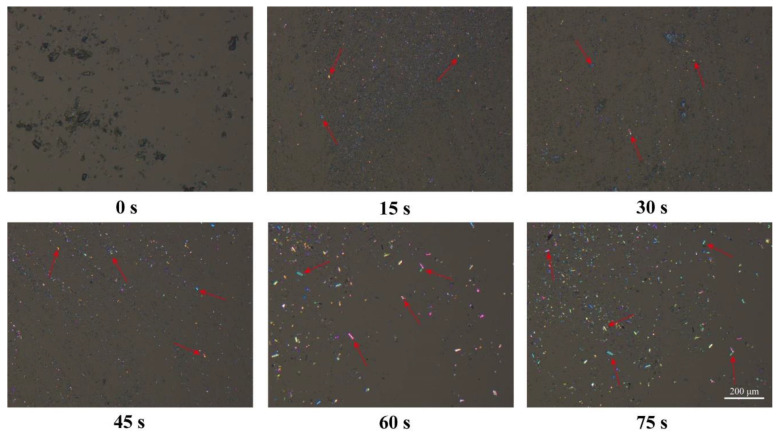
PLM images of amorphous ACM in water at different points (red arrows indicate birefringence).

Figure 2 shows the PLM images of amorphous ACM at various time points in water. The fresh amorphous ACM showed no birefringence under PLM. However, birefringence was observed after hydration for 15 s, suggesting that the amorphous ACM recrystallized upon coming into contact with water. Moreover, ACM crystals grew rapidly over time, and rod-shaped crystals were clearly observed after hydration for 60 s. Therefore, it was reasonable to speculate that the crystallization tendency and gel formation might be the cause of the slow dissolution of AXM.

### 3.3. DSC

**Figure 3:**
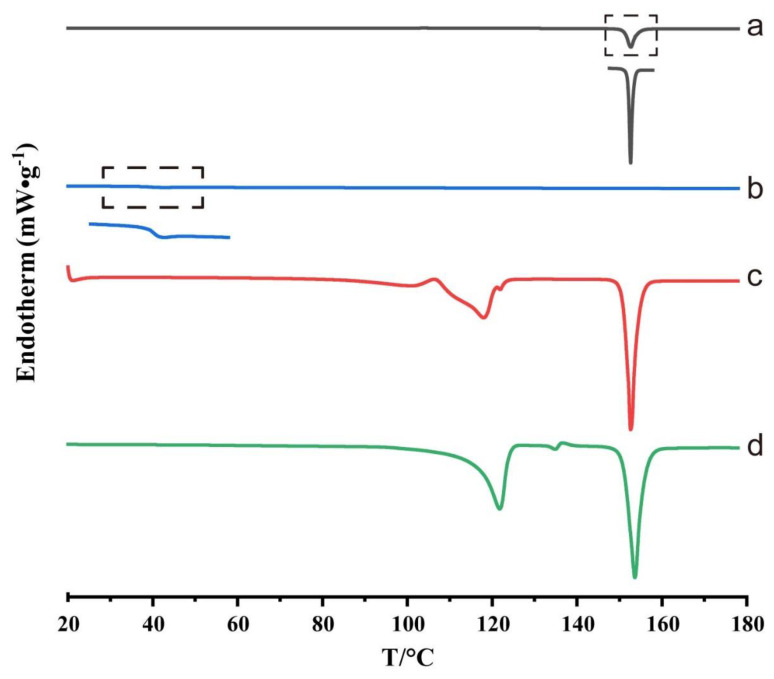
DSC thermograms of (**a**) crystalline ACM, (**b**) amorphous ACM, (**c**) monohydrate ACM and (**d**) ACM gels.

DSC curves of crystalline ACM, amorphous ACM and ACM gels are shown in Figure 3. Crystalline ACM showed an obvious endothermic peak at 152.7 °C, which corresponded to its melting point (Figure 3a). Amorphous ACM did not show any peaks but a Tg at 39.2 °C (Figure 3b). Monohydrate ACM exhibited a melting peak at 153 °C, along with another endothermic peak at 121 °C due to the dehydration [18]. As shown in Figure 3d, the ACM gels showed similar thermal behaviors to monohydrate ACM, suggesting that recrystallization had occurred in the gel formation and that the majority of the formed gels were monohydrate ACM.

### 3.4. XRPD

**Figure 4:**
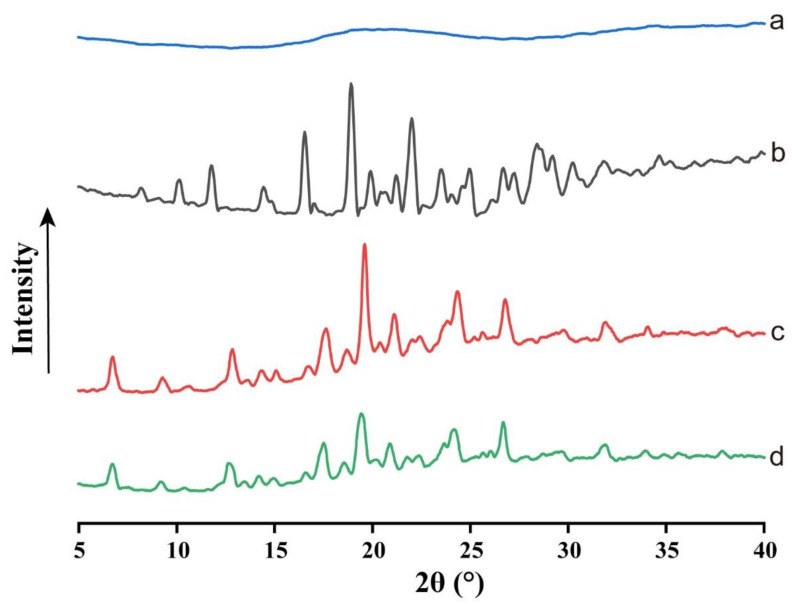
XRPD patterns of (**a**) amorphous ACM, (**b**) crystalline ACM, (**c**) monohydrate ACM and (**d**) ACM gels.

XRPD was performed to confirm the solid state of the ACM gels. The XRPD patterns of crystalline ACM, amorphous ACM, monohydrate ACM and ACM gels are shown in Figure 4. Crystalline ACM exhibited characteristic diffraction peaks at 2θ of 8.19°, 10.13°, 11.76°, 14.49°, 16.54°, 18.90°, 21.21°, 23.52°, 24.94°, 26.67° and 28.41°, which was consistent with the relevant reports [13]. However, only a broad halo was observed in amorphous ACM. For monohydrate ACM, it displayed characteristic diffraction peaks at 2θ of 6.74°, 9.28°, 12.84°, 14.38°, 16.74°, 17.62°, 19.59°, 21.10°, 24.36° and 26.79°, as previously reported [18]. The formed gels showed same characteristic diffraction peaks with monohydrate ACM, indicating that recrystallization to monohydrate ACM during the dissolution. This result was consistent with DSC analysis.

### 3.5. FTIR

**Figure 5:**
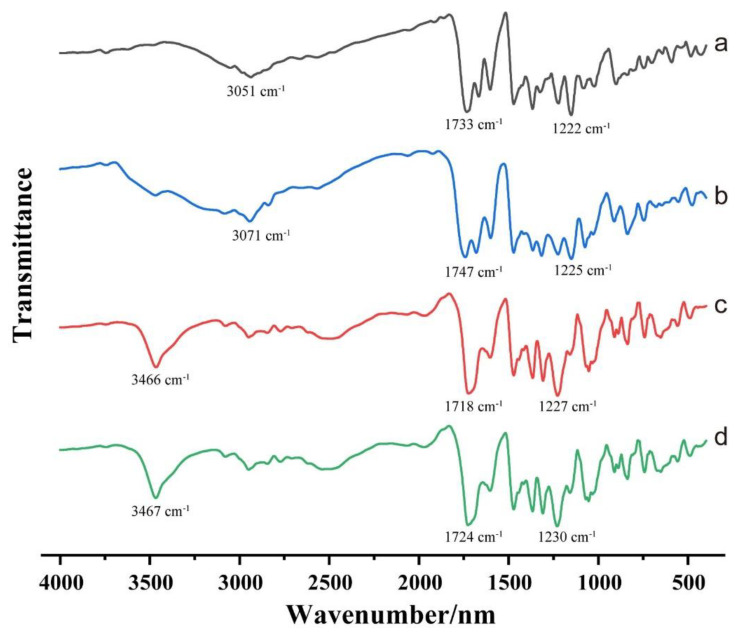
FT-IR spectra of (**a**) crystalline ACM, (**b**) amorphous ACM, (**c**) monohydrate ACM and (**d**) ACM gels.

As shown in Figure 5, crystalline ACM showed its typical characteristic peaks at 3051 cm^−1^, 1733 cm^−1^ and 1222 cm^−1^, which were attributed to the stretching vibration of carboxylic OH, C=O and C−O, respectively (Figure 5a). Compared with crystalline ACM, the amorphous ACM showed the characteristic peaks mentioned above but at 3071 cm^−1^, 1747 cm^−1^ and 1225 cm^−1^ (Figure 5b). These changes might be attributed to the disruption of structured crystal lattice into disordered structure and reconfiguration of ACM molecular [19]. For the ACM gels and the monohydrate ACM, their FT-IR spectra overlapped (Figure 5c,d), and they showed an absorption peak of O-H stretching vibration at 3466 cm^−1^ due to the presence of crystal water [18,20]. This confirmed that the amorphous ACM recrystallized to a monohydrate during the dissolution due to the polar glycolic ester group [13].

### 3.6. Influencing Factors of Amorphous ACM Gelation

**Figure 6:**
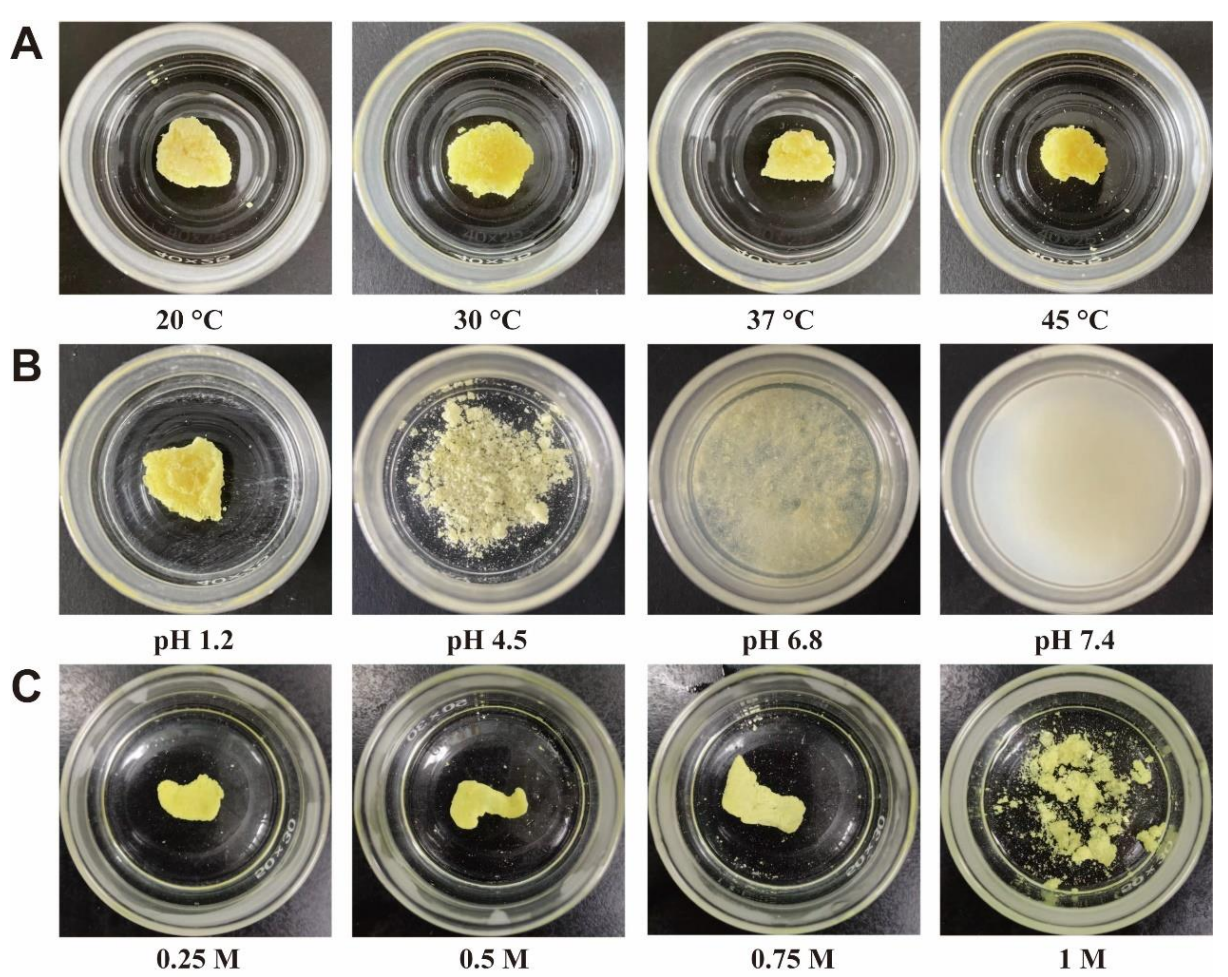
Images of amorphous ACM in a beaker after dissolution in aqueous solutions at different (**A**) temperatures, (**B**) pH values, and (**C**) ionic strengths.

The amorphous ACM was immersed in water at different temperatures (20, 30, 37 and 45 °C), and the images of ACM gels are shown in Figure 6A. Amorphous ACM floated on the surface upon contacting with water, followed by gradual aggregation and formation of a viscous gel block at 20 °C. Similar gelation phenomenon was also observed at 30, 37 and 45 °C. However, the amorphous powders aggregated and formed a gel more easily at a higher temperature due to the increased molecular mobility [21]. In addition, when the temperature was near or above Tg, amorphous ACM transformed into the high-viscosity supercooled state to promote gel formation [11,22].

In addition to the temperature, this study also investigated the effect of pH value on gelling performances of the amorphous ACM. As shown in Figure 6B, the formation of gel is dependent on the pH value. The amorphous ACM was able to aggregate into a yellow gel block in the hydrochloric acid solutions at pH 1.2. However, when the pH value was increased to 4.5, a small number of soft clumps were observed and no complete gel blocks were formed. Moreover, yellow-colored suspensions, rather than gels, were formed at pH 6.8 and 7.4, and obvious stratification was observed after standing. Since ACM is a weakly acidic drug [23], recrystallization is difficult as the pH value increases. The absence of gelator reduced gel formation capacity.

Salt ions were inevitably introduced when evaluating the pH effect, so it was necessary to investigate the effect of ionic strength on the gels. As the ionic strength increased from 0.25 to 0.75 M, the gelation time increased (Table S1). Moreover, amorphous ACM could not even form a complete gel at a salt concentration of 1 M (Figure 6C), demonstrating that too many ions inhibited gelation. The gels are difficult to form at a high ionic strength, because the number of water molecules interacting with ACM decreases due to ion competition [24].

### 3.7. Texture Profile Analysis

**Figure 7:**
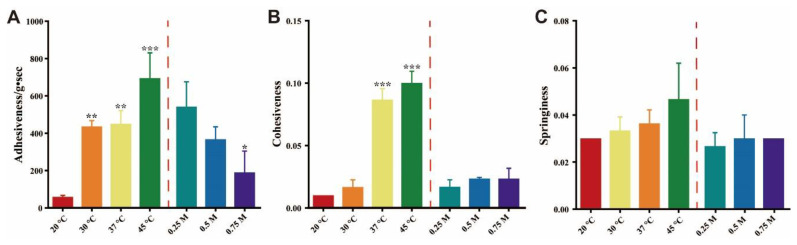
Texture parameters: (**A**) adhesiveness, (**B**) cohesiveness and (**C**) springiness of the ACM gels from amorphous ACM at different temperatures and ionic strengths. Results are expressed as the mean ± SD (*n* = 3, * *p* < 0.05, ** *p* < 0.01 and *** *p* < 0.001 vs. 20 °C in temperature effect experiments or vs. 0.25 M in ionic strength effect experiments).

In order to further quantitively analyze the effects of various factors (temperatures and ionic strengths) on the ACM gels, the gelling parameters (adhesiveness, cohesiveness and springiness) of the ACM gels were measured using a texture analyzer. Adhesion is a combined effect of cohesive and adhesive forces [25]. As shown in Figure 7A, the adhesion of the ACM gel at 45 °C was significantly enhanced 11.96-fold compared with the gels formed at 20 °C. As the temperature increased, the amorphous ACM showed significant sticky properties because it switched to the supercooled state [9]. This might be the internal reason for the slow dissolution of the amorphous ACM. In addition, the gel adhesion decreased dramatically with increasing ionic strength. This might be because the addition of other ions reduced the interaction of ACM with water molecules and thus inhibited gel formation [26].

Cohesiveness represents the internal strength of a material and is defined as the degree to which it can be deformed before rupture. Springiness is the rate at which a deformed material returns to its original state after compression [27]. These two parameters are indicators measuring the difficulty of breaking the gels [22]. The cohesiveness of ACM gels improved significantly by 0.09 and the springiness increased slightly by 0.01 with the temperature increased from 20 °C to 45 °C (Figure 7B,C). This suggested that the internal structure of ACM gels is difficult to disrupt at a relatively high temperature. The increased temperature enhanced the molecular mobility, accelerated the nucleation rate and promoted the crystallization of ACM [28]. Increased crystallization might increase the compactness and hardness of ACM gels, thus causing the gel to become difficult to destroy. However, the cohesiveness and springiness of ACM gels did not show significant difference under different ionic strengths.

### 3.8. Gelation Mechanism Analysis

From the results obtained, it can be concluded that amorphous ACM lost its dissolution advantage due to the gelation. Further characterization demonstrated that the ACM gel was composed of recrystallized monohydrate ACM. Thus, recrystallization was proved to be the internal reason of gelation, while hydration also promoted the formation of gels.

Gels are colloidal dispersions that consist of the components including the liquid dispersion medium and the gelator [29,30]. Therefore, two conditions must be met for gelation, including the presence of the gelator and its interaction with the solvent [31]. It has been reported that crystallization acts as a gelator for polymer gelation [32,33], while crystallization of a small molecule can also drive the gelation. Yin et al. reported that cefotaxime sodium crystallized into nano- and micron-sized crystals, and the crystals then aggregated and adsorbed solvents to induce gelation [34].

**Figure 8:**
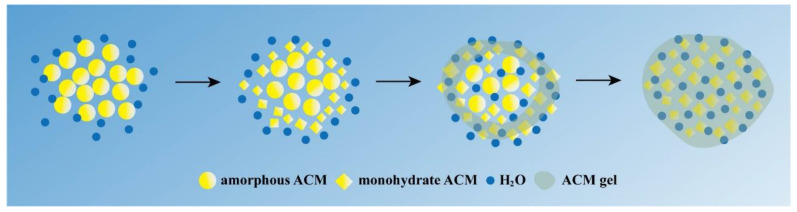
Schematic diagram of gel formation from amorphous ACM.

In our study, the characterizations by PLM, DSC, XRPD and FTIR revealed the presence of the crystals in the ACM gels as well, which proved to be the monohydrate ACM (Figure 2, Figure 3, Figure 4, Figure 5). Therefore, it was reasonable to speculate that the recrystallization might be the driving force for the formation of ACM gels (Figure 8). As a metastable system, amorphous drugs have high surface free energy due to the lack of periodic arrangement, which theoretically increases the surface area and enhances molecular mobility [35,36]. Therefore, ACM molecules readily hydrated when contacting with water. Since the solubility of monohydrate ACM is lower than that of the anhydrous form [18], monohydrate is more easily recrystallized, and these formed crystals aggregated and captured water molecules to form a gel. As shown in dissolution tests (Figure 1), amorphous ACM powders contacting with water would form a supersaturated state with high viscosity and then recrystallize. These fine crystals combine to form a “film” around the amorphous core. With paddle stirring, the internal amorphous ACM that have not yet contacted with water were wrapped by the recrystallized film to form an agglomerate. As time went on, water gradually penetrated into the agglomerate, and the internal amorphous ACM was recrystallized to form a gel. Therefore, the formed ACM gels would hinder the penetration of the medium into the ACM and inhibit the diffusion of the dissolved drug into the bulk solution, resulting in its slow dissolution.

This mechanism was further proved by the evaluation of the influencing factors of amorphous ACM gelation. As shown in Figure 6 and Figure 7, the increase in pH inhibited the formation of the gelator and hindered gel formation. With respect to ionic strength, when it increased, the interaction of the solvent with the gelator was inhibited due to ion competition, leading to a consequent decrease in the gelling capacity of the amorphous ACM. The above experiments demonstrated that recrystallization and hydration are indispensable for gel formation. In addition, the experiments carried out at different temperatures suggested that the supercooled state theory plays a major role when the temperature is around or above Tg, while the recrystallisation to fine crystals theory better explains the gelation mechanism at lower temperatures. Both of them can explain the formation of amorphous drug gels and complement each other.

We elucidated a possible new mechanism for the gelation of amorphous drugs, which provides a new idea for the exploration of amorphous drug gels. This will help to the inhibition of amorphous drug gelation by applying pharmaceutical techniques. Han et al. reported that the gelation of amorphous curcumin could be eliminated by the addition of hydrophilic excipients [37]. The subsequent study has shown that preparing co-amorphous forms had the same effect [10]. These studies provided favorable and promising strategies to eliminate gelation for amorphous materials, overcome poor dissolution behaviors and enhance physical stability. On the other hand, it is possible to exploit the gelation properties to develop sustained-release formulations.

## 4. Conclusions

This study reported that amorphous ACM showed a significantly lower dissolution than the crystalline ACM due to the gelation of amorphous ACM. The further characterizations of the ACM gels suggested that they may be formed by recrystallization to fine crystals and adsorption of water molecules. This new mechanism is further confirmed as a supplement to the supercooled state theory by evaluation of the influencing factors of amorphous ACM gelation. In summary, this study replenishes the research orientation of amorphous drugs gel formation and provides new ideas for the development of poorly water-soluble drugs.

## Data Availability

The data presented in this study are available on request from the corresponding author.

## Supplementary Materials

The following are available online at https://www.mdpi.com/article/10.3390/pharmaceutics15010219/s1, Table S1: The gelation time of amorphous ACM under different condition.
