# Supplementary material for: Recrystallization Mediates the Gelation of Amorphous Drugs: The Case of Acemetacin
Source: Pharmaceutics. 2023 Jan 8;15(1):219. doi: 10.3390/pharmaceutics15010219 (PMC9860709; doi:10.3390/pharmaceutics15010219)
Supplement: Supplementary file 1 [file pharmaceutics-15-00219-s001.zip › pharmaceutics-2108746-supplementary.pdf]

Table. S1. The gelation time of amorphous ACM under different condition. Data are mean ± SD. (n=3, ***$p < 0.001$ *vs* 20°C in temperature effect experiments or *vs* 0.25 M in ionic strength effect experiments)

Under different conditions		Gelation time (s)
Temperature (°C)	20	6.53 ± 0.14
	30	4.79 ± 0.09***
	37	4.03 ± 0.11***
	45	3.21 ± 0.18***
pH values	1.2	5.45 ± 0.20
	4.5	–
	6.8	–
	7.4	–
Ionic strength (M)	0.25	5.73 ± 0.32
	0.5	6.31 ± 0.17
	0.75	7.84 ± 0.44***
	1.0	–

"–" referred to no gelation time
